# Perceptual averaging on relevant and irrelevant featural dimensions

**DOI:** 10.3758/s13414-024-03005-2

**Published:** 2025-01-10

**Authors:** Philip T. Quinlan, Dale J. Cohen, Keith Allen

**Affiliations:** 1 Department of Psychology, The University of York, Heslington, York, YO10 5DD UK; 2 Department of Psychology, University of North Carolina at Wilmington, Wilmington, NC USA; 3 Department of Philosophy, University of York, York, UK

**Keywords:** Perceptual averaging, Ensemble coding, Ensemble perception

## Abstract

Here we report four experiments that explore the nature of perceptual averaging. We examine the evidence that participants recover and store a representation of the mean value of a set of perceptual features that are distributed across the optic array. The extant evidence shows that participants are particularly accurate in estimating the relevant mean value, but we ask whether this might be due to processes that reflect assessing featural similarity rather than computing an average. We set out and test detailed predictions that can be used to adjudicate between these averaging and similarity hypotheses. In each experiment, a memory display of randomly positioned bars was briefly presented followed immediately by a probe bar. Participants had to report in a Yes/No task whether the probed feature value was present. In initial experiments, we examine reports of the orientation of white bars and of the color of vertical bars. Then, in companion experiments, we examine reports of the orientation of bars whose color vary, and of the color of bars whose orientation varies. In this way, we test ideas about whether perceptual averaging occurs on a featural dimension that is irrelevant to the task. Currently, it is not known whether perceptual averaging only takes place on a task-relevant dimension or whether it operates more widely.

**Supplementary information:**

The online version contains supplementary material available at 10.3758/s13414-024-03005-2.

## Introduction

Ensemble coding refers to the human visual system’s apparent ability to rapidly recover global statistical information about the content of the immediate optic array. For example, if randomly positioned texture elements are distributed throughout the visual field, an observer is able to recover a reasonably accurate estimate of the average orientation of those elements even if the presentation of the array is brief (i.e., no more than 500 ms; see, e.g., Dakin & Watt, 1997; Parkes et al., 2001). Although there is mounting evidence that such perceptual averaging can take place on a variety of featural dimensions (see the recent review by Whitney & Yamanashi Leib, 2018), some crucial issues remain. In Experiments 1 and 2, we pit two competing explanations of the data against one another: (1) the traditional explanation that the data reflect a process of recovering and operating upon a representation that codes the never presented mean featural value (Ariely, 2001; Jeong & Chong, 2020; Watamaniuk & Duchon, 1992), or (2) the alternative explanation that the data reflect processes that are sensitive to the similarity of a probe to the *actually presented* feature values (cf. Myczek & Simons, 2008). In Experiments 3 and 4, we assess the degree to which perceptual averaging reflects attentional processing (see Chen et al., 2021, for a recent example) by testing whether perceptual averaging takes place on a dimension that accompanies the judged dimension, but is irrelevant to the task.

Much of the extant evidence for perceptual averaging comes from studies in which participants are asked to estimate the mean value of presented features on a particular perceptual dimension such as the size of a circle or orientation of a line (e.g., Khayat & Hochstein, 2018). Whereas this procedure can show the degree to which participants can extract the mean value, it provides little information about the underlying processes that make that extraction possible. A more direct method of looking at these processes is provided by the Yes/No task (Ariely, 2001; Rajendran et al., 2020). Consider a key experiment by Ariely (2001) in which a “memory” display, containing several circles that differed in size, was presented for 500 ms. Immediately following, participants were asked to judge whether a single probe circle had been present in the memory display (i.e., respond Yes or No). The data from two participants were plotted as a function of the number of circle sizes present in the memory display and the number of circles in the display. No statistical tests are reported, but visual inspection of the respective curves indicates that the participants’ tendency to accept a novel item as present varied as a function of its distance from the mean size *regardless* of whether it had actually been present. In a separate experiment, Ariely showed that participants “encode quite precise information about the mean” of sizes of the set of circles. (p. 201). Ariely (2001) concluded that the overriding propensity is to recover global attributes of a given feature set and discard information about individual items in the set: mean feature value is recovered and stored, but not the actual presented feature values.

Supporting evidence for this view has been reported in a particularly relevant follow-up study by Khayat and Hochstein (2018). In their experiments and, on each trial, participants viewed RSVP displays in which a sequence of 12 centrally presented items unfolded over time. Sequences comprised circles of various sizes, bars of various orientations, or circles that varied in gray-level. Each item was presented for 100 ms separated by a blank interval of 100 ms. At the end of the sequence the participant was presented with two probe items and was instructed to choose which had been present in the display. Positive probes were either an old item that possessed the mean featural value of the items that had been presented or another old item. An item that possessed the mean value was known as an *Amean* item, an item that possessed a feature value within the range of those presented was known as an *A probe*, and a *B probe* was one that possessed a value outside the range of those presented. Whereas B probes were never presented in the displays, Amean and A probes may either have been presented or not. A general finding was that memory for old Amean probes was better than for old A probes and, more interestingly, accuracy of report scaled inversely with a probe’s distance from the mean (cf. Ariely, 2001). In addition, for new probe trials, participants were more accurate in rejecting the probe the further the probe was from the mean.

Amongst other things, such data as these were taken to support the view that participants automatically recovered the mean featural value of the presented items – a view that accords well with Ariely’s (2001). However, in neither case is the evidence definitive because there are two competing hypotheses that can predict that the mean value of a display will be identified more often as present than the actual presented items themselves. We call these, respectively, the *Perceptual Averaging Hypothesis* and the *Similarity Hypothesis*. The extant data are generally assumed to fit most comfortably with the former hypothesis, but the latter hypothesis has never been ruled out. That is, the tendency to report the mean may arise not because of ensemble encoding that eventuates in a representation of the mean, but because the Yes/No task reflects processes that are sensitive to the similarity of the probe to the old items (where similarity is defined as distance to the nearest displayed item). By definition, because the mean probe is more similar on average to a random memory item than is any other probe, it may be identified as present even more so than any old item.

Here, we replicate and extend the findings of Ariely (2001) and pit the two competing hypotheses against one another. Our memory display contained a randomly positioned array of 64 oriented bars (with a random half of the bars of one orientation and the remaining half of another). Participants judged whether the designated feature of a probe bar was present in the display. In the first experiment, all the bars were presented in white (half of the bars were of one orientation and half were of another) and the participant had to judge whether the orientation of a probed bar was present in the memory display. The probe was either the mean of these two orientations (i.e., a mean or M probe), one of these two orientations (i.e., an old or O probe), or a new orientation that falls outside the range of the two orientations in the memory display (i.e., a novel or N probe). Assume that the bars on a trial are sampled from a series of seven possible items (numbered 1–7). Two such items will be selected for inclusion in the memory display. Also assume, for expository convenience, that half of the sampled bars are “1s” then the remaining bars will be “3s”. The M probe will be a “2” but the N probe can be a “4,” “5,” “6,” or “7.” Once the displayed bars are chosen then the M probe is fixed but the N probe is randomly selected from the remaining series items. By systematically varying the distance of the N probes, we aim to adjudicate between the two hypotheses.

**Fig. 1:**
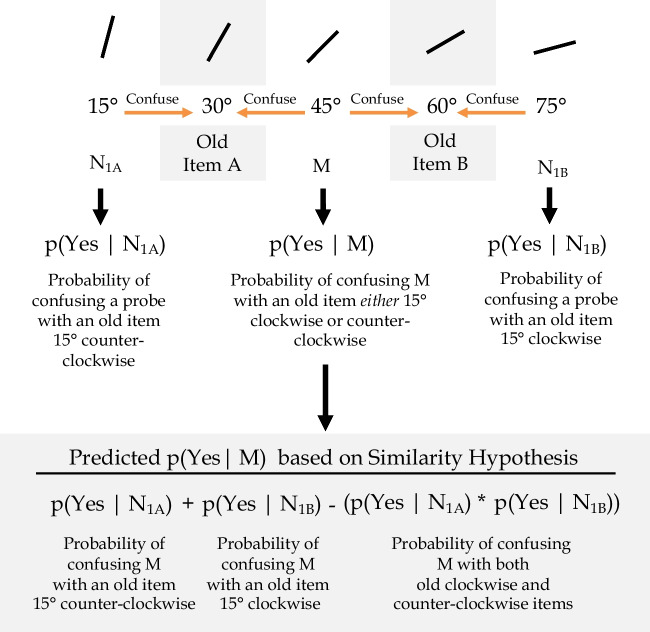
How predictions are derived from the Similarity Hypothesis

According to the Perceptual Averaging Hypothesis, the probability of responding “No” should increase directly with its distance from the mean. In addition, the mean item should be the item most erroneously categorized as being present. The Similarity Hypothesis often makes the same prediction. However, in contrast to the Perceptual Averaging Hypothesis, in our experiment the Similarity Hypothesis makes a very precise point prediction about the probability of falsely reporting the M probe as present, *p(*Yes|*M*) (see Fig. 1). For this analysis, some of the N probes are critical because they are the *same* distance from the old items as the M probe. We will call these N probes, N_1A_ and N_1B_. The Similarity Hypothesis assumes that these probes are independent and as confusable with the old items as is the M probe. As such, the probability of reporting these probes as present, *p(*Yes|N_1A_) and *p(*Yes|N_1B_), provide a measure of the confusability of the M probe with the old items. If the Similarity Hypothesis is correct, the probability of confusing the M probe with the old items, *p(*Yes|*M*), should equal the joint probability of confusing either the N_1A_ probe and/or the N_1B_ probe with the old items (see Fig. 1):

1 $$p{(\text{Yes}|M)}_{\text{similarity }}= p\left(\text{Yes}|{\text{N}}_{1\text{A}}\right)+ p\left(\text{Yes}|{\text{N}}_{1\text{B}}\right)- [p(\text{Yes}|{\text{N}}_{1\text{A}}) \times p(\text{Yes}|{\text{N}}_{1\text{B}})]$$

or, equivalently,

2 $$p{(\text{No}|M)}_{\text{similarity }}= p(\text{No}|{\text{N}}_{1\text{A}}) \times p(\text{No}|{\text{N}}_{1\text{B}})]$$

The first row illustrates the orientation probes. Below them is an illustration of where confusions may occur between the new probes and the old probes. Below that illustrates how the predictions arise from the data. For expository convenience, N probes here as shown as being 15° away from the nearest old item. In the experiment proper the distance between a N probe and the nearest old item varied according to which items had been selected as the old items and what the remaining items were in the series. Given these constraints, on a given trial the N probe was selected at random from the remaining items in the series (see text for further details)

**Fig. 2:**
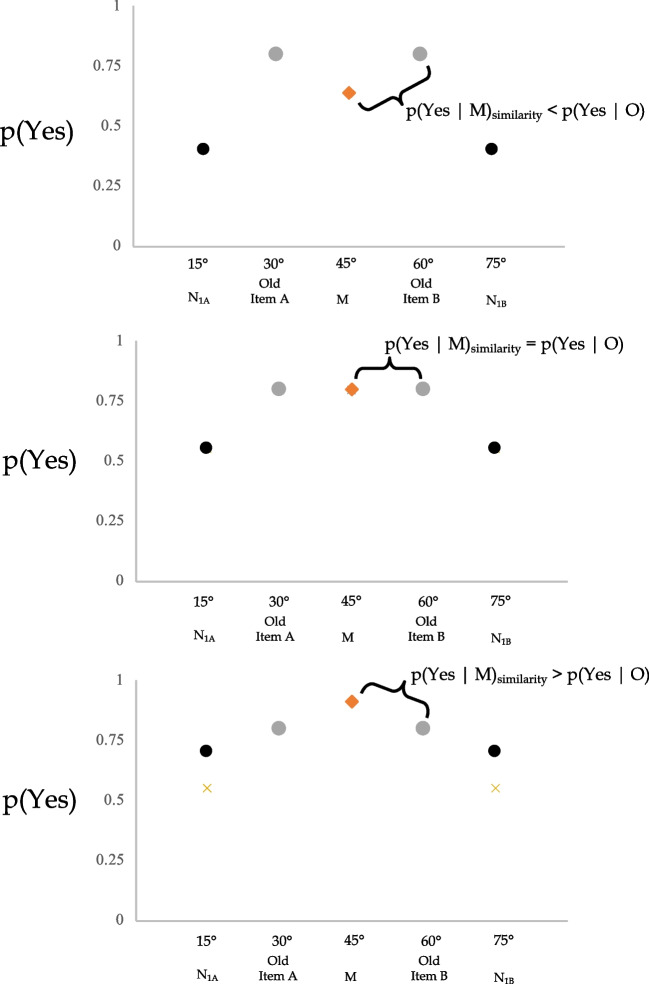
Three hypothetical patterns of data predicted by the Similarity Hypothesis. In all graphs *p(*Yes|M)_similarity_ is presented in red, *p(*Yes|O) is presented in gray, and *p(*Yes|N_1_) is presented in black. *p(*Yes|O) remains constant in all graphs. We varied *p(*Yes|N_1_) and calculated *p(*Yes|M)_similarity_. The top graph illustrates a pattern whereby *p(*Yes|M)_similarity_ < *p(*Yes|O), the middle graph illustrates a pattern whereby *p(*Yes|M)_similarity_ = *p(*Yes|O), and the bottom graph illustrates a pattern whereby *p(*Yes|M)_similarity_ > *p(*Yes|O)

Figure 2 illustrates three hypothetical patterns of results, where *p(*Yes|*M*)_similarity_ < *p(*Yes| O), *p(*Yes|*M*)_similarity_ = *p(*Yes| O), and *p(*Yes|*M*)_similarity_ > *p(*Yes| O). Because the Similarity Hypothesis can potentially predict all three of these outcomes (depending on the relative detectability of the old probes), it cannot be assumed that Perceptual Averaging is driving the data when *p(*Yes|*M*)_similarity_ > *p(*Yes| O). Rather, it has to be established whether or not the data can be explained by the Similarity Hypothesis. Specifically, if *p(*Yes|*M*)_similarity_ = *p(*Yes|*M*), then the Similarity Hypothesis cannot be ruled out. However, if *p(*Yes|*M*)_similarity_ < *p(*Yes|*M*), then there is strong evidence for the Perceptual Averaging Hypothesis.

Evidence from a companion experiment (Experiment 2) will be used again to try to adjudicate between these two hypotheses but this time in relation to color. In Experiment 2 all the bars in the memory display were vertically oriented, but they were colored and varied in lightness. In this regard, the experiment is a partial replication of that reported by Rajendran et al. (2020), but, in this case, it acted as a means to contrast the Perceptual Averaging and Similarity Hypotheses.

Finally, Experiments 3 and 4 address the degree to which ensemble encoding depends on attentional control. As Chen et al. (2021) have argued, the evidence is somewhat mixed on this question (see, e.g., the interchange between Myczek and Simons (2008) and Chong et al. (2008)). Currently, the bulk of the evidence is restricted to cases where participants were instructed to make judgments about “attended” features on one dimension (such as the orientation of line segments; see, e.g., Chen et al. 2021), and then effects of attention are gauged via the degree to which performance is affected by other “unattended” features on the same dimension. Here we examine the question of whether ensemble coding takes place on an otherwise irrelevant featural dimension. If ensemble encoding takes place automatically on all features in an array, then information from the irrelevant dimension should influence judgments on the relevant dimension and vice versa. This will be reflected in whether or not participants are seduced into committing erroneous “Yes” responses if the value on the irrelevant dimension is the mean. In Experiment 3, participants judged color when the orientation of the memory items also varied across trials and, in Experiment 4, participants judged orientation when the color of the memory items also varied across trials.

## Experiment 1: Judge orientation/constant color

In Experiment 1, we assess whether the Perceptual Averaging hypothesis or the Perceptual similarity hypothesis best predicts probe detection when the relevant dimension is the orientation of bars. Here, color will be held constant (white, xyY - [0.273, 0.301, 29.392], L*ab – [110.404, −10.672, −42.851]).

### Methods

#### Participants

Participants were recruited via the Department of Psychology Participants panel at the University of York. The panel predominantly comprises students at the University of York. Participants were recruited via the SONA participant on-line booking tool and were offered a small payment or course credit (where appropriate) as recompense. Participants fitted the following inclusion criteria: aged between 19 and 40 years, have normal or corrected-to-normal vision, and not have any color vision deficits.

To determine the sample size, we ran a power simulation. Specifically, for each participant, we simulated the binomial distribution for each condition, assuming the response probabilities from a pilot study (in which PQ and KA acted as participants). We repeated this for groups of participants sized two to ten, in steps of one. We then fit our models to the data and noted the BIC and *r*^*2*^ of each model fit. We determined the best fit model by the model with the lower BIC. We bootstrapped this simulation 500 times for each sample size. The results showed that we reached power of over .95 for the weakest test with ten participants averaging 12 trials per condition (where condition was defined as steps from the mean on N probe trials). To ensure an adequate sample size, we ran 25 participants in the first experiment (more than double the number estimated by the power analysis).

#### Design, stimuli, and equipment

The experimental task was based on the Yes/No task described by Ariely (2001). On a given trial, a memory display was presented briefly (for 500 ms) followed by a probe display. The memory display comprised 64 randomly positioned bars and the probe display contained a single centrally presented bar. The participant was instructed to judge whether the critical feature of the probe bar had been present in the memory display and respond accordingly, either “Yes” or “No.”

The experiment ran in a web browser via an iiyama Vision Master 505 21-in. color monitor. The screen resolution was set at 1,600 × 1,200 and the refresh rate was set at 60 Hz. The bars, each 40 pixels in length 6 pixels wide, were displayed in an 800 (high) × 1,000 (wide) pixel centrally positioned region. None of the memory bars were displayed within a circular region of the center (with a radius equal to the length of a bar) so as to avoid any superimposition of a memory bar with the subsequently presented central probe bar. The background color of the screen was set to gray (#808080, xyY - [0.303, 0.328, 20.234], L*ab – [52.101, −2.774, −1.286]). The screen was gamma corrected via the use of the DataColor SpyderX Elite package and the colorimetry was undertaken with these tools.

In Experiment 1 (the *Judge orientation/constant color experiment*), all of the bars were presented in white, but half of the memory bars were of one orientation and half were of another. The orientations to be presented were sampled from 0° to 90° separated by 15° steps. On a random half of the trials, the bars were defined relative to a rightward tilt and on the remaining half of the trials the bars were defined relative to a leftward tilt. Prior to a given trial, two orientations from the sample were selected at random such that the chosen orientations were 30° apart. On half the trials (on 240 trials) the probe bar was one of these old items (an O probe) and the remaining trials were evenly divided between M probes (i.e., the mean of the old orientations) and N probes (i.e., the orientation was more extreme than the old orientations). On N trials the probe was selected at random from the remaining possible orientations excluding the old items and the mean. On each trial, the participant had to decide whether the probe orientation matched either of the old orientations.

#### Procedure

On each trial a small central white dot acted as an initial central fixation point presented for 500 ms. This was immediately followed by the presentation of the memory display for 500 ms. At the offset of the memory display, the probe display was presented until response. A Yes response was assigned to the “K” key and a No response was assigned to the “D” key.

Although reaction times (RTs) were automatically collected and participants were instructed to respond as quickly and as accurately as they could, RTs were not analyzed. Following Ariely (2001), sole interest is with the nature of the response on every trial and whether this signified a “Yes” or “No” response. It is not accuracy per se that is of critical interest but whether the tendency to respond “No” varies according to type of probe.

The experimental scripts were written in Javascript and called the relevant jsPsych libraries (de Leeuw, 2015). For each experiment, an initial block of 20 practice trials was presented (data from these trials were discarded prior to analysis) followed by five blocks of 96 experimental trials.

Participants were tested individually in a small, darkened testing room containing a PC computer, monitor, keyboard, and mouse. Participants sat facing the monitor at a distance of approximately 60 cm. Responses were collected via keyboard keypresses.

### Results and discussion

One participant was excluded because they were at chance with N probes that are maximally distant from the mean. The primary aim was to test the competing hypotheses. For the *Perceptual Averaging* tests, the probe trials were divided up according to the probe’s distance from the mean. For the *Similarity* tests the probe trials were divided up according to the probe’s distance from the nearest old item. The data were cast as the proportion of No responses for each probe type.

To assess the Similarity Hypothesis, for each participant, we calculated *p(*No|M) directly from the data and the predicted *p(*No|M)_similarity_ using the N_1_ probes. These data were then entered into a paired-sample *t*-test so as to determine the relation between *p(*No|M)_similarity_ and *p(*No|M). If *p(*No|M)_similarity_ = *p(*No|M) then the Similarity hypothesis is strongly supported. In contrast, if *p(*No|M)_similarity_ > *p(*No|M) then the Perceptual Averaging Hypothesis is strongly supported. If *p(*No|M)_similarity_ < *p(*No|M) then there is strong evidence against the Perceptual Averaging Hypothesis. This result is also inconsistent with a strong form of the Similarity hypothesis that assumes that the N1 items are perceptually independent.

**Fig. 3:**
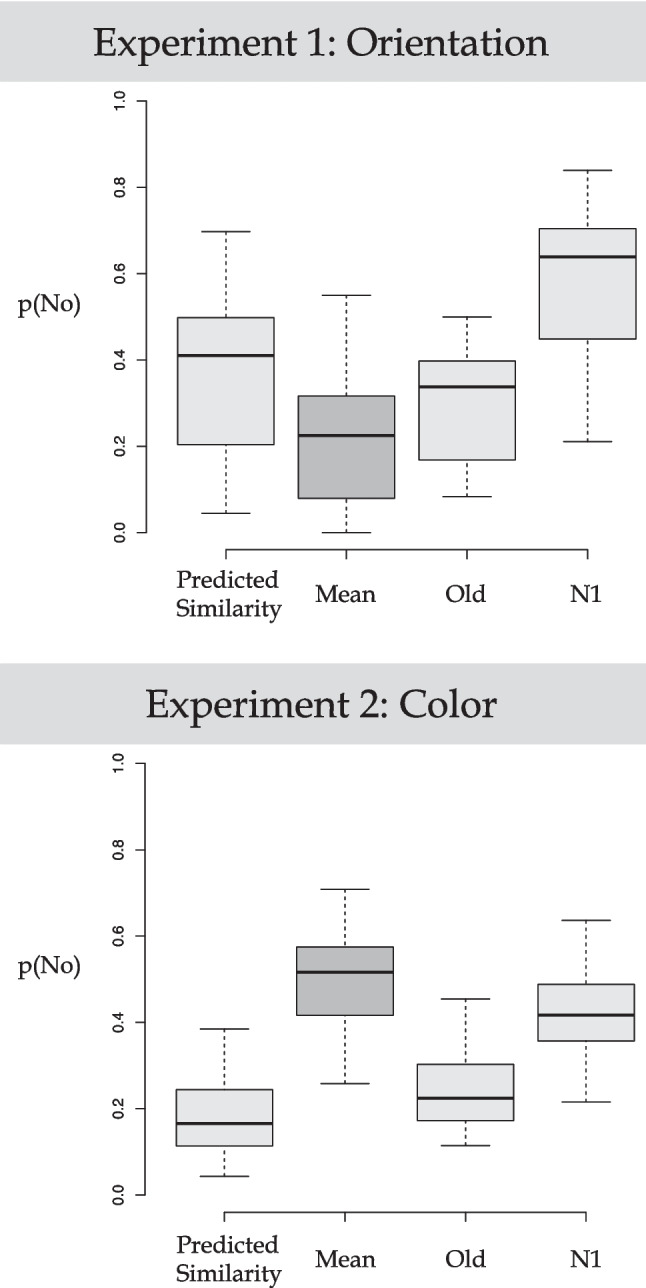
Boxplots of the data of interest in Experiment 1: Judge orientation/constant color (top panel) and Experiment 2: Judge color/constant orientation (bottom panel). Mean, Old, and N1 refer to the probe types and Predicted Similarity refers to estimates of *p(*No) computed from Eq. 2

Figure 3 top panel shows boxplots displaying *p(*No|M)_similarity_, *p(*No|M), *p(*No|Old), and *p(*No|N1) averaged over all participants. The average *p(*No|M) was 0.217 (SD = 0.16) and the *p(*No|M)_similarity_ was 0.372 (SD = 0.11). A paired *t*-test revealed that the corresponding difference was statistically reliable, *t*(23) = −4.87, *p* < .001, *d* = 0.994, BF_10_ = 403.590 (i.e., decisive evidence against Ho, Wetzels et al., 2011). Participants were more likely to accept the mean orientation as having been present than predicted by the Similarity Hypothesis. We also ran a repeated-measures one-way ANOVA comparing *p(*No|M), *p(*No|N1), *p(*No|Old). Table 1 gives the summary statistics of the conditions of interest. There was a significant effect of probe, *F*(2, 46) = 127.6, *p* < 0.001. A post hoc analysis (Tukey) revealed that *p(*No|N1) > *p(*No|Old) > *p(*No|M), all *p*s < .01. This evidence supports the Perceptual Averaging Hypothesis. Notably, participants were more likely to accept the mean orientation as having been present than other orientations that actually had been present.

**Table 1:** The *p*(No) estimated marginal means of and standard error in parentheses for each condition in which the irrelevant dimension was constant

			Probe	
	Target dimension	Mean	Old	N1
Experiment 1	Orientation	0.22 (0.03)	0.30 (0.03)	0.58 (0.03)
Experiment 2	Color	0.50 (0.02)	0.24 (0.02)	0.42 (0.02)

We also compared how well the data are fit by the following log-logistic function when scored according to two contrasting classificatory schemes,

3 $$p{\left(No|M\right)}_{similarity}=a+ \frac{(1-a)}{1+ {e}^{(b-cx)}}$$

Here *a* and *b* and *c* are free parameters, and *x* is the ordinal distance of the probe either from the mean (so as to test the perceptual averaging account) or the nearest old item (so as to test the similarity account). For each experiment the two data fits were compared using BIC.

We simply note here that Eq. 3 is equivalent to:

4 $$p{\left(Yes|M\right)}_{similarity}=\frac{(1-a)}{1+ {e}^{(b-cx)}}$$

**Fig. 4:**
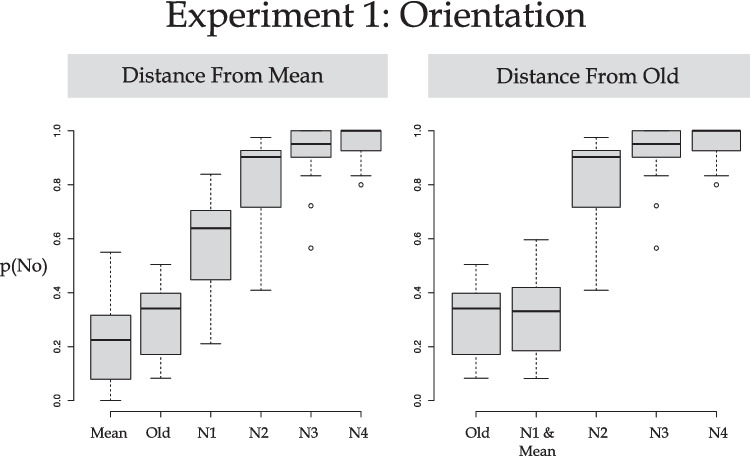
Boxplots of the data interest in Experiment 1: Judge orientation/constant color broken down according to probe type: N1–4 reflects distance from an old item. The N1 & Mean indicates the data include both N1 and Mean probe trials as both are equidistant from the Old items

Each participants’ data were fit with the log-logistic function when scored according to the averaging account (distance from Mean) and, separately, the similarity account (distance from Old). These data are plotted in Fig. 4. Visual analysis of Fig. 4 reveals a smooth ogive when the data are plotted as a function of the distance from Mean. In contrast, there appears to be a threshold effect when plotted as the distance from Old. Despite the visual support of the Perceptual Averaging hypothesis, the average BIC for the averaging account was −20.038 and for the similarity account it was −21.135. These measures of fit were shown not to be statistically different, *t*(23) = 0.31, *p* = 0.762, d = 0.06, BF_10_ = 0.224 (i.e., substantial evidence in favor of H0). Both accounts fit the data very well (average *r*^*2*^ for the averaging account = .995, *r*^*2*^ for the similarity account = .993).

In sum, although the log-logistic data fitting failed to provide discriminatory evidence, the comparison of *p(*No|M) and *p(*No|M)_similarity_ provides strong support for the Perceptual Averaging hypothesis when relevant dimension is the orientation of bars.

## Experiment 2: Judge color/constant orientation

In Experiment 2, we assess whether the Perceptual Averaging hypothesis or the Perceptual Similarity hypothesis best predicts probe detection when the relevant dimension is the color of bars. Here, orientation will be held constant (vertical).

### Methods

#### Participants

The same participants’ exclusion criteria from Experiment 1 were applied in Experiment 2. Although we attempted to recruit 25 participants, one participant was mis-assigned to the other color judgment experiment (Experiment 3) hence our final sample was 24 participants.

#### Materials

In Experiment 2 (the *Judge color/constant orientation* experiment), all of the bars were vertical. However, now the luminance of the bars varied in a manner consistent with the orientation manipulation in Experiment 1. Two series of seven colors were used, one green, one blue. Across each series chromaticity was maintained (*x* and *y* were kept constant) but luminance (Y) varied (see chromaticity diagrams in the Appendix Figs. 6, 7, 8 and 9). Table 2 provides details of the two series of colors.

**Table 2:** The green and blue color series and their corresponding parameter specifications

x	y	Y	L	a	B	R	G	B	Hex
Green series									
0.322	0.498	10.759	39.172	−28.659	29.259	62	105	40	#3E6928
0.324	0.496	16.090	47.092	−31.958	33.359	77	128	51	#4D8033
0.326	0.492	23.511	55.595	−34.968	37.306	95	155	63	#5F9B3F
0.325	0.488	32.637	63.866	−36.799	38.568	113	184	76	#71B84C
0.323	0.491	47.588	74.564	−45.063	46.344	132	213	90	#84D55A
0.320	0.493	60.685	82.209	−50.536	50.183	146	236	100	#92EC64
0.320	0.492	70.950	87.461	−52.972	52.558	158	254	108	#9EFE6C
Blue series									
0.220	0.188	1.416	12.064	8.684	−20.551	25	30	61	#191E3D
0.217	0.189	6.141	29.765	12.824	−33.442	58	67	124	#3A437C
0.219	0.194	10.006	37.853	13.698	−37.276	74	85	155	#4A559B
0.221	0.198	14.833	45.405	14.564	−41.460	90	103	185	#5A67B9
0.219	0.195	20.207	52.070	16.783	−47.263	105	120	214	#6978D6
0.218	0.193	25.237	57.306	18.712	−51.811	118	135	239	#7687EF
0.218	0.193	28.784	60.591	19.551	−54.133	126	143	253	#7E8FFD

x, y, and Y measures reflect xyY parameters. L is defined in CIE-L*ab space. The RGB values are sRGB 0–255 values and HEX shows the corresponding HTML values. The colorimetry was undertaken via the DataColor SpyderX Elite package

Half the memory bars were chosen to be of one luminance and half of another with the two luminance values separated by one intervening value (that defined the mean). On half the trials the bars were green and on half they were blue. On each trial the participant had to decide whether the probe color matched either of the old colors.^1^

#### Procedure

The procedure in Experiment 2 was identical to that of Experiment 1.

### Results and discussion

The data from Experiment 2 followed the same processing pipeline as Experiment 1. Two participants were removed for performing worse than chance with probes that were maximally distant from the mean.

Figure 3 bottom panel shows boxplots displaying *p(*No|M)_similarity_, *p(*No|M), *p(*No|Old), and *p(*No|N1) averaged over all participants. The average *p(*No|M) was 0.492 (SD = 0.11) and the *p(*No|M)_similarity_ was 0.181 (SD = 0.09). A paired *t*-test revealed that the corresponding difference was statistically different, *t*(21) = 12.35, *p* < .001, d = 2.63, BF_10_ = 2.109 x 10^+8^ (i.e., decisive evidence against H0). Participants were less likely to accept the Mean as being present than predicted by the Similarity Hypothesis. We also ran a repeated-measures one-way ANOVA comparing *p(*No|M), *p(*No|N1), *p(*No|Old); see Table 1 for summary statistics of the conditions of interest. There was a significant effect of probe, *F*(2, 42) = 57.87, *p* < 0.001. Post hoc analyses (Tukey) revealed that *p(*No|M) > *p(*No|N1) > *p(*No|Old), all *p*s < .01. This is strong evidence against the Perceptual Averaging account when the relevant dimension is color. Given that *p(*No|M) > *p(*No|N1), this indicates that the mean probe is *less* similar to the Old items than are the N1 items.

**Fig. 5:**
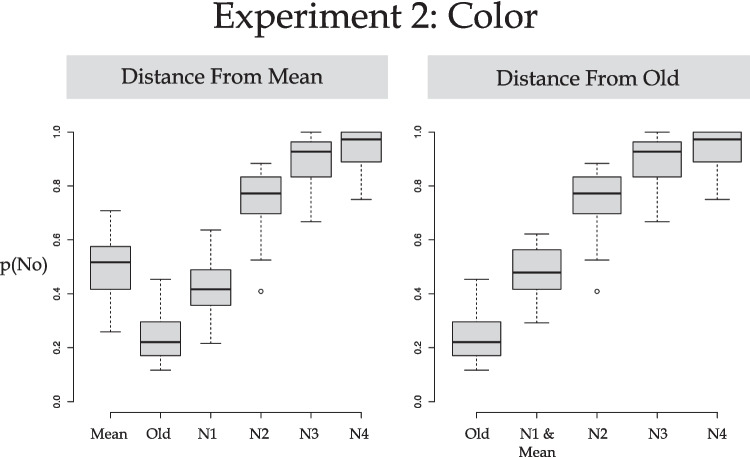
Boxplots of the data interest in Experiment 2: Judge color/constant orientation broken down according to probe type; N1–4 reflects distance from an old item

The data were also fit with a log-logistic function when scored according to the Perceptual Averaging account, and separately for the Similarity account. These data are plotted in Fig. 5. Visual analysis of Fig. 5 reveals a smooth ogive when the data are plotted as a function of the distance from Old (right panel). In contrast, when plotted as a function of distance from Mean there is an apparent discontinuity (left panel). Visual inspection supports the Perceptual Similarity hypothesis and this is confirmed by the model fit statistics. The average BIC for the averaging account was −3.857 and for the similarity account it was −11.180. These measures of fit were shown to be statistically different, *t*(21) = 3.603, *p* = .00167, d = 0.77, BF_10_ = 22.895 (i.e., strong evidence against H0). This shows that the better fit was provided by the Similarity account than the Perceptual Averaging account even though both accounts fit the data very well (average *r*^*2*^ for the Averaging account = .930, *r*^*2*^ for the Similarity account = .970). In sum, the more detailed data fitting provided evidence that it was the similarity of the probe’s luminance to an old probe’s luminance that had a significant effect upon responding. There is no evidence that perceptual averaging of color, and specifically luminance, took place.

## Experiment 3: Judge color/varying orientation

The data from Experiments 1 and 2 were clear: perceptual averaging took place when judging orientation, but not when judging color. In Experiment 3, we assess whether the perceptual averaging of orientation occurs even when orientation is *irrelevant* to the judgment. If it does, then this strongly suggests that perceptual averaging of orientation is an automated and perhaps “compulsory” process (Parkes et al., 2001).

To test this, we replicated Experiment 2, such that participants again judged the presence of the color of the probe, whilst the orientation of the displayed items varied as in Experiment 1. We assume that recognition of the probe’s color will be influenced by the irrelevant nature of its orientation. If perceptual averaging of orientation is automatic, then a probe with the irrelevant Mean orientation will influence the recognition in the same way as a probe with an irrelevant Old orientation. If Perceptual Similarity is automatic, then a probe with the irrelevant Mean orientation will influence the recognition in the same way as a probe with an irrelevant N1 orientation.

### Method

#### Participants

The same participants’ exclusion criteria from Experiment 1 were applied here. Given that a participant was mis-assigned from the other color judgment experiment (Experiment 2), the eventual sample contained 26 participants.

#### Materials

In this experiment (i.e., the *Judge color/varying orientation* experiment), both orientation and color (luminance) of the probe varied even though participants were instructed to merely judge whether the color of the probe was present in the memory display. Now the trials were arranged according to factorial combinations of the manipulation of orientation (M, N, O) and the manipulation of color (M, N, O). On the O (color) probe trials there were 80 for each of the M, N, and O orientation probes, and on the M and N (color) probe trials there were 40 for each of the M, N. and O orientation probes.

#### Procedure

The procedure in Experiment 3 was identical to that of Experiment 2.

### Results and discussion

Three participants were removed for performance worse than chance for probes that were maximally distant from the mean. Because we varied the irrelevant orientation dimension, and we expect that variation to influence color processing, it cannot be considered a pure assessment of color processing. We therefore will consider only how variation on the irrelevant orientation dimension influences color processing. In our registered report, we proposed a 3 (relevant dimension: M, O, N) × 3 (irrelevant dimension: M, O, N) repeated-measure analysis on *p(*No). However, collapsing all the N trials into a single category (N1 – N4) makes the N trials more dissimilar to the Old trials than to the Mean trials. We therefore conducted the above analysis with only the N1 trials included. That is, we calculated a 3 (relevant color: M, O, N1) × 3 (irrelevant orientation: M, O, N1) repeated-measure analysis on *p(*No) (see Table 3 for summary statistics of the data of interest). The originally proposed analysis can be found in the Online Supplementary Materials.

**Table 3:** The *p*(No) estimated marginal means of and (SE) for each condition for each level of the irrelevant dimension

				Probe	
Experiment	Relevant dimension	Irrelevant dimension	Mean	Old	N1
3	Color	Orientation			
		Mean	0.44 (0.03)	0.18 (0.02)	0.43 (0.04)
		Old	0.45 (0.03)	0.20 (0.02)	0.42 (0.04)
		N1	0.50 (0.03)	0.24 (0.02)	0.49 (0.04)
4	Orientation	Color			
		Mean	0.26 (0.03)	0.37 (0.03)	0.60 (0.05)
		Old	0.24 (0.03)	0.31 (0.03)	0.54 (0.05)
		N1	0.35 (0.03)	0.40 (0.03)	0.52 (0.05)

There was a significant main effect of relevant color probe information, *F*(2, 44) = 47.53, *p* < .001, *p(*No|M)_relevant_ = 0.461, *p(*No|N1)_relevant_ = 0.444, *p(*No|Old) _relevant_ = 0.206. Post hoc analyses (Tukey, p < 0.05) of relevant color revealed that *p(*No|Mean)_relevant_ = *p(*No|N1)_relevant_ > *p(*No|Old)_relevant_. This finding replicates the conclusions of Experiment 2: The Mean probe was identified as being present less often than the Old probe and about as often as the N1 probe.

There was also a significant main effect of irrelevant orientation information, *F*(2, 44) = 3.49, *p* = 0.039: *p(*No|M)_irrelevant_ = 0.348, *p(*No|N1)_irrelevant_ = 0.409, *p(*No|Old)_irrelevant_ = 0.355. Post hoc analysis (Tukey) of this main effect revealed that participants were most likely to respond absent to the probe if its irrelevant orientation was new (i.e., N1), *p* = 0.053, for the comparison with Mean_irrelevant_ trials, and, *p* = 0.090 for the comparison with Old_irrelevant_ trials. Participants were equally likely to respond absent to the probe if its orientation was the Mean or Old, *p* > .05. This pattern of data, *p(*No|Mean)_irrelevant_ = *p(*No|Old)_irrelevant_ > *p(*No|N1)_irrelevant_, supports the conclusion that perceptual averaging is occurring on the irrelevant orientation dimension and this does influence detection of the probe’s color. The corresponding two-way interaction failed to reach statistical significance, *F* < 1.0.

## Experiment 4: Judge orientation/varying color

In Experiment 1, we showed that perceptual averaging does occur on the orientation of the displayed bars when this is the relevant dimension. The data from Experiment 3 revealed that perceptual averaging of orientation also occurs when this dimension is irrelevant to the judgment. In Experiment 2, we showed that perceptual similarity was driving responses when the color of bars was the relevant dimension. In Experiment 4, we assess whether perceptual similarity or perceptual averaging of the color of the items influences judgments of the probe’s orientation.

### Method

#### Participants

The same participants’ exclusion criteria from before were applied here. We recruited 25 participants.

#### Materials

In Experiment 4 (the *Judge orientation/varying color* experiment), both orientation and color (luminance) of the probe varied even though participants were instructed to judge only whether the orientation of the probe was preset in the memory display. Again, the trials were arranged around factorial combinations of the manipulation of the orientation (M, N, O) and the manipulation of the color (M, N, O). On the O (orientation) probe trials there were 80 for each of the M, N, and O color probes, and on the M and N (orientation) probe trials there were 40 for each of the M, N, and O color probes.

### Results and discussion

We analyzed the data in Experiment 4 using the same analysis pipeline as Experiment 3. Three participants were removed in the current case for performance worse than chance for probes that were maximally distant from the mean. The *p(*No) scores were entered into a 3 (relevant orientation: M, O, N1) × 3 (irrelevant color: M, O, N1) repeated-measures ANOVA (see Table 3 for the summary statistics of the data of interest). There was a significant main effect of relevant orientation information, *F*(2, 42) = 59.46, *p* < .001, *p(*No|N1)_relevant_ = 0.555, *p(*No|Old)_relevant_ = .360), *p(*No|Mean)_relevant_ = 0.282). Post hoc analyses (Tukey) of this relevant orientation main effect revealed that *p(*No|N1) > *p(*No|Old) > *p(*No|Mean), all *p*s < .05. This pattern, *p(*No|N1) > *p(*No|Old) > *p(*No|Mean), replicates a central result of Experiment 1, namely, that the Mean probe was identified as being present more often than were the actual Old probes.

There was a significant main effect of irrelevant color information, *F*(2, 42) = 4.96, *p* = .012, *p(*No|Old)_irrelevant_ = 0.362, *p(*No|N1)_irrelevant_ = 0.424, *p(*No|Mean)_irrelevant_ = 0.412. Post hoc analyses (Tukey) of this irrelevant color main effect revealed that participants were least likely to classify the probes as being absent when the probe’s color was Old relative to when it was either New (*p* = .053) or the Mean (*p* = .0135). There was no difference in absent reports when the probe’s color was New or the Mean, *p*> .05. This pattern of data, *p(*No|Mean)_irrelevant_ = *p(*No|N1)_irrelevant_ > *p(*No|Old)_irrelevant_, support the conclusion that perceptual similarity is taken into account on the irrelevant color dimension and influences the detection of the probe’s orientation.

These results are tempered by a significant two-way interaction between relevant and irrelevant probe information, *F*(4, 84) = 3.39, *p* = .013. When the relevant probe’s orientation was the Mean, participants were most likely to respond absent when the probe’s color was N1 relative to when the probe’s color was the Mean or when it was Old, both *p*s < .05. There was no difference in absent responding for the latter two cases, *p* > .05. Thus, the pattern was *p(*No|N1)_irrelevant_ > *p(*No|Mean)_irrelevant_ = *p(*No|Old)_irrelevant_. However, when the orientation of the probe was Old, participants were least likely to respond absent when the color of the probe was Old relative to when it was New or the Mean, both *p*s < .05. Participants were equally likely to respond absent in the latter two cases, *p* > 05. The pattern was *p(*No|N1)_irrelevant_ = *p(*No|Mean)_irrelevant_ > *p(*No|Old)_irrelevant_.

These data reveal an interesting pattern. When the relevant orientation of the probe is the Mean, the irrelevant color of the probe appears to influence orientation detection consistent with perceptually averaging (the Mean and Old irrelevant dimensions have equal influence). However, when the probe is the Old orientation, the irrelevant color of the probe appears to influence orientation detection consistent with the Perceptual Similarity Hypothesis (the Mean and N1 have equal influence).

## General discussion

The initial questions that have been addressed here concern pitting a Perceptual Averaging account of probe detection against a Perceptual Similarity account. The key question is which account provides the best explanation of how information is extracted and used from brief displays comprising many oriented colored bars. In Experiment 1 participants judged whether the orientation of a single probe bar had been present in the display that contained only white bars and performance clearly showed that performance was well explained by the perceptual averaging hypothesis. A key finding was that participants were more likely to accept the mean orientation as having been present than the Old probe as predicted by the Perceptual Similarity hypothesis. This evidence is consistent with the claim (cf. e.g., Ariely, 2001) that a representation of the average orientation is actually recovered and operated on during item recall: The mean orientation is treated as though it was actually present in the display.

In Experiment 2 participants were asked to judge whether the color of a probe had been present in the display that contained only vertical bars. Again, to avoid any misunderstanding, we varied “color” by varying luminance for a given hue. When we varied “color,” bars of the same hue were presented that differed in luminance, so although we varied hue across trials, within trials it was luminance differences that were key. On these grounds, our discussion of color processing reflects differences in luminance and not chromaticity. In Experiment 2 there was some evidence that participants were less likely to accept the Mean luminance as having been present relative to the N1 colors but the more detailed analyses revealed that performance was best explained by how similar the probe’s color was to an old item. There was no evidence that a representation of the mean color (luminance) was recovered and operated on during item recall. Thus, for color processing, probe discrimination is best explained by the Perceptual Similarity hypothesis.

Experiments 3 and 4 were conducted to assess whether perceptual averaging (or perceptual similarity) manifested on the irrelevant dimension. If it does, then it likely operates automatically on the dimension in question. In Experiment 3 the relevant dimension was color, but we also varied the irrelevant dimension (orientation). In this case participants were overall as likely to reject the Mean and the N1 colors as being present (replicating Experiment 2), but now judgments of color were also influenced by the (irrelevant) orientation of the probe. Here the data were relatively clear cut in showing evidence of perceptual averaging of orientation. Participants were less likely to accept the probe as having been present if its orientation was an N1 orientation than if it was the Mean or an Old orientation. Here again therefore is evidence that the mean orientation of the displayed bars was recovered and implicated in item recall. This is further evidence of perceptual averaging of orientation information and in this case the evidence is that such averaging takes place even when orientation is irrelevant to the judgment being made.

A more complex picture emerges from the data in Experiment 4. Here participants judged orientation when color varied irrelevantly. Now it was found that the influence of color varied according to what the probe’s orientation was. Firstly, if the probe’s orientation was an N1 orientation, then judgments of orientation were unaffected by the nature of the probe’s color. When the probe’s orientation was the Mean, the influence of the irrelevant color dimension was equivalent when the probe was the Mean and the Old. If the color (luminance) of the probe was new (i.e., N1), then participants responded absent more often relative to the other kinds of probes. However, a quite different pattern emerged when the probe’s orientation was Old. Now the influence of the irrelevant color was equivalent when the probe was the Mean and the N1. If the color of the probe was Old, then participants responded absent less often relative to the other kinds of probes.

Overall, therefore, something that emerges very strongly in the present results is that judgments of orientation and those of color reflect the operation of very different forms of perceptual processing. Judgments of orientation reflect operations involving perceptual averaging. Notably irrelevant orientation information also influenced judgments of color consistent with perceptual averaging. In sum, there is evidence of perceptual averaging of orientation taking place regardless of whether or not it is relevant to the task. On these grounds it seems as though that perceptual averaging of orientation is compulsory (Parkes et al., 2001) and may well reflect something basic about texture perception.

The current data are far less clear about the kind of processing that take place when participants judged color. When there is no variation in the irrelevant orientation dimension, then color clearly reveals a pattern consistent with processing being sensitive to the similarity relations between the bars’ colors, and specifically luminance. When color was the irrelevant dimension, the pattern of perceptual similarity held when the relevant probe orientation was Old. In contrast, when the relevant probe orientation was the Mean, then the irrelevant color dimension showed some evidence of perceptual averaging of the old luminance values. This is a quite unexpected and surprising finding.

Given the strong evidence of the similarity-based processing of color in the data, the evidence of perceptual averaging in Experiment 4 clearly stands out as being distinctive and discordant. In this regard the current work has failed to provide a very clear picture of what kind of ensemble processing takes place when color is the irrelevant dimension. This is perhaps not so surprising given the complexities of the nature of color. In this regard, Rajendran et al. (2020) found evidence for different kinds of ensemble coding for chromaticity and luminance. They argued that ensemble coding for stimuli differing in chromaticity may occur within colour categories, but not across large hue differences. In contrast to this, they claim that luminance may represent a more continuous perceptual dimension. In addition, although they suggest that there is a possibility that a metrical average is computed for luminance, we found very little evidence for this. We therefore suspect a quite different account of luminance processing is needed: one that takes into account the similarity relations that exist across a display.

In conclusion, the present findings provide strong evidence for perceptual averaging of orientation regardless of whether orientation is relevant to the task or not. In contrast it seems that judgments of color/luminance depend more on similarity relations to the actual displayed colors. The degree to which perceptual averaging takes place more generally remains for future work to establish. Finally, the fact that irrelevant variation of orientation influenced color judgments and irrelevant variation of color influenced orientation judgments provides further evidence for the non-independence of the processing of color and form information (cf. Cohen, 1997). The co-dependencies between these dimensions appears to be as important to study as are any within-dimension characteristics that may otherwise be observed.

## Electronic supplementary material

Below is the link to the electronic supplementary material.

Supplementary file1 (PDF 63 KB)

## Figures and Tables

**Fig. 6:**
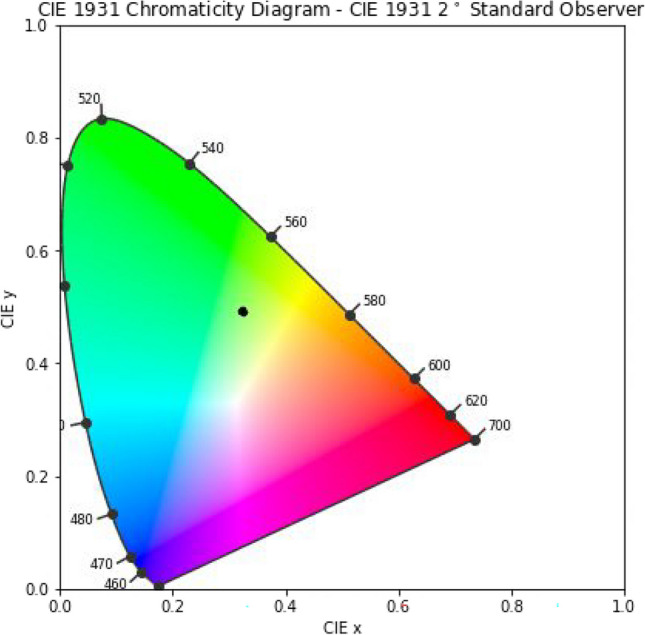
Chromaticity diagram for the items in the green series. Seven black dots are plotted to show the location of each item in the series in this color space. Most of the dots are superimposed

**Fig. 7:**
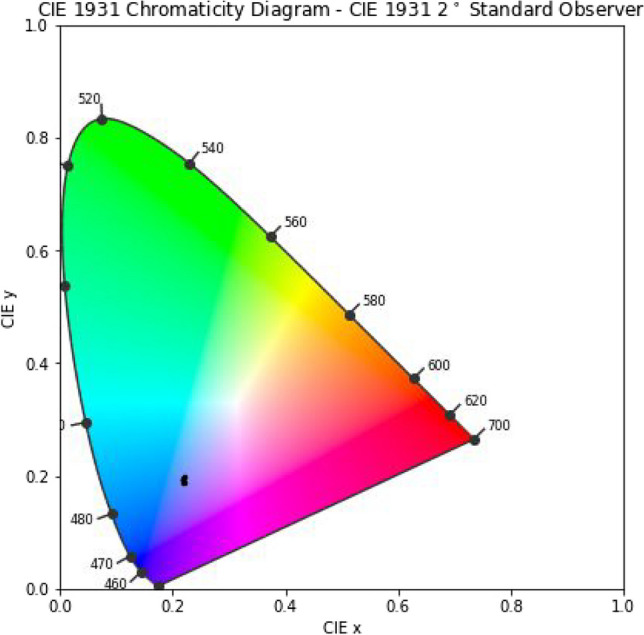
Chromaticity diagram for the items in the green series. Seven black dots are plotted to show the location of each item in the series in this color space. Most of the dots are superimposed

**Fig. 8:**
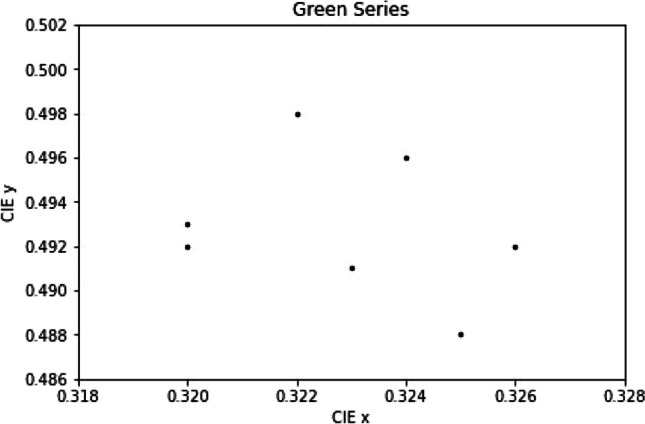
Magnified chromaticity diagram for the items in the green series

**Fig. 9:**
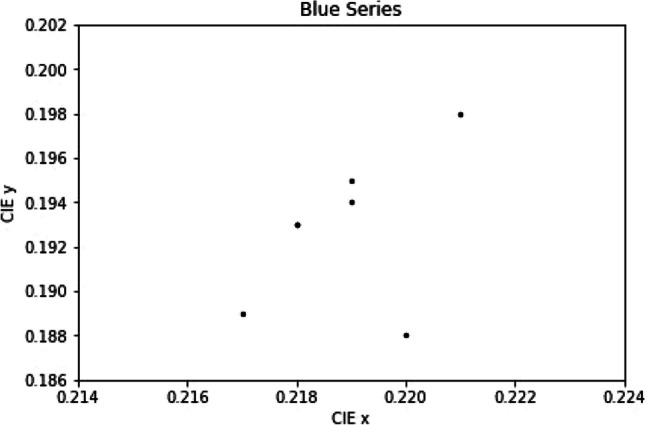
Magnified chromaticity diagram for the items in the blue series. *Note*. Only six dots are visible as two are superimposed

## Data Availability

Raw data files and scripts for analysis are available at: https://github.com/ccpluncw/ccpl_data_ec2021.git

## Footnotes

1 To be clear, at a general level our interests lie with color processing, but in order to systematize variation in this domain changes in luminance were enacted (see Table 2). Strictly speaking therefore, it is variation in luminance and not chromaticity that is key. However, to make the task understandable to participants, reference to “color” and not “luminance” was included in the task instructions. In the following reference is also made to “color” on the understanding that the critical manipulation is with respect to luminance.

## Code availability

For queries regarding the experimental scripts please contact the first author.
