# Supplementary material for: Perceptual averaging on relevant and irrelevant featural dimensions
Source: Atten Percept Psychophys. 2025 Jan 10;87(2):698–711. doi: 10.3758/s13414-024-03005-2 (PMC11865180; doi:10.3758/s13414-024-03005-2)
Supplement: Supplementary file 1 — Supplementary file1 (PDF 63 KB) [file 13414_2024_3005_MOESM1_ESM.pdf]

Supplementary Materials

Experiment 3

The p(No) scores were entered into a two-way repeated measures ANOVA in which the fixed factors were relevant probe information (Color relevant: Mean, Old, or New), irrelevant probe information (Orientation irrelevant: Mean, Old, New). Only the main effects of relevant probe information, $F(2, 44) = 132.6$, $p < .001$, and irrelevant probe information $F(2, 44) = 4.907$, $p < .001$, were statistically reliable. The corresponding two-way interaction failed to reach statistical significance, $F(4, 88) = 1.06$, $p = .381$

Decomposing the main effect of relevant color information, it was found that participants were most likely to accept an Old probe color as being present (p(No) = .221), were comparatively less likely to accept a Mean color probe as being present (p(No) = .461) and least likely to accept a New color as being present (p(No) = 0.643) (all ps < .001). This again accords with the idea that it is similarity processes that are entrained rather than averaging processes when judging color. In turn, decomposing the main effect of irrelevant orientation information, it was found that participants were generally less likely to judge a probe as having been present if its orientation was New (p(No) = .483) than if it was the Mean (p(No) = .414), $p = .013$, or Old (p(No) = 0.429), $p = 0.06$. Participants were equally likely to reject a color probe if its orientation was either the Mean or Old, $p = .809$. This is in line with the evidence of perceptual averaging of orientation information. Critically though such averaging takes place when orientation is irrelevant to the task.

Experiment 4

The p(No) scores were entered into a 3 (orientation relevant: M, O, N) x 3 (color irrelevant: M, O, N) repeated measures ANOVA. The main effects of relevant probe information, $F(2, 42) = 177.2$, $p < .001$, and irrelevant probe information, $F(2, 42) = 18.15$, $p < .001$, were both statistically reliable, as was the corresponding two-way interaction, $F(4, 84) = 11.42$, $p < .001$. The main effect of relevant probe information came about because participants were least likely to respond absent for the Mean orientation (p(No) = .308), more likely to respond absent for the Old orientations (p(No) = .391) and most likely to respond absent for the New orientations (p(No) = .735), all $p < .003$. The main effect of irrelevant probe information arose because participants were most likely to respond absent to the probe if its color was New, p(No) = .562, than if the color was the Mean, p(No) = .452, or Old, p(No) = .420, $p < .001$. The difference between Mean and Old color probes did not reach statistical difference, $p = .420$.

To examine the two-way interaction in further detail, pairwise comparisons were carried separately for the data broken down according to relevant probe information. When the relevant probe was Mean, participants were more inclined to respond absent when the probe color was New (p(No) = 0.426) than when it was either Old (p(No) = 0.235), $p < .001$, or the Mean color (p(No) = 0.261), $p < .001$. Participants were equally likely to respond absent when the probe's color was the Mean or that of an Old item, $p = .737$. Exactly the same pattern of performance was found when the relevant probe was an Old orientation – participants were most inclined to respond absent when the probe color was New (p(No) = 0.491) than if its color was the Mean (p(No) = 0.371), or of an Old color (p(No) = 0.310), both ps < .001. As before, participants

were equally likely to respond present when the probe's color was the Mean or that of an Old item, $p = .072$. However, when the relevant probe information specified a New orientation responses were unaffected by the nature of the probe's color (all *p*s > .05).
